# Impact of the COVID-19 pandemic on well-being and quality of life of patients with alpha-1-antitrypsin deficiency

**DOI:** 10.1186/s12931-023-02553-9

**Published:** 2023-10-25

**Authors:** Catherina Werdecker, Robert Bals

**Affiliations:** 1 Department of Internal Medicine V – Pulmonology, Allergology and Critical Care Medicine, Saarland University, Kirrberger Strasse 1, 66421 Homburg/Saar, Germany; 2 Molecular Therapies for Lung Disease, Helmholtz-Institute for Pharmaceutical Research Saarland – HIPS, D-66123 Saarbrücken, Germany

## Abstract

**Background:**

Alpha-1-antitrypsin deficiency (AATD) is a genetic disorder characterized by mutations in the SERPINA1 gene, primarily affecting the lungs and liver. The COVID-19 pandemic has raised questions about the susceptibility of individuals with AATD to COVID-19 and whether patients with rare lung disease might experience increased stress-related symptoms and mental health challenges. This study aims to investigate the impact of the COVID-19 pandemic on the quality of life of individuals living with AATD.

**Methods:**

The study enrolled participants from the German registry for individuals with AATD. Questionnaires were sent to the 1250 participants, and a total of 358 patients were included in the analysis. The primary objective was to examine the influence of sociodemographic and disease-related factors on the occurrence of stress-related symptoms. This was accomplished through correlation and regression analyses. We also investigated the role of baseline quality of life (QoL), as measured by the St. George’s Respiratory Questionnaire (SGRQ), as a mediator of this relationship.

**Results:**

Stress-related symptoms were predicted by young age, female gender, psychological disorders, and a history of exacerbations of lung disease, as determined by multiple regression analysis. QoL as measured by the SGRQ mediated the relationship between poor lung function, stress, and a decline in overall well-being.

**Conclusion:**

The presented data demonstrate that the COVID-19 pandemic significantly affects the psychological well-being of patients with rare diseases, leading to increased levels of anxiety and stress. Disease-related factors can exacerbate stress manifestations, especially when compounded by sociodemographic and contextual factors. Thus, our study emphasizes the crucial role of taking these factors into account when managing individuals with AATD in pandemic situations.

## Introduction

Alpha-1-antitrypsin deficiency (AATD) is a genetic disorder that results in a reduced concentration of alpha-1-antitrypsin (AAT) in the body due to a mutation in the SERPINA1 gene [1, 2]. AAT plays a crucial role in regulating inflammatory reactions in the lungs and preventing tissue damage caused by proteases. AAT deficiency, particularly in chronic smokers, can lead to the early onset of lung diseases such as chronic obstructive pulmonary disease (COPD) or emphysema [3, 4]. AATD can also cause liver diseases [5, 6] and rare conditions such as inflammation of the subcutaneous fat (panniculitis) [7] or vasculitis [8].

Patients with AATD face a particular challenge during the early months of the COVID-19 pandemic as it was speculated that they might have an increased risk of developing severe disease outcomes if infected with SARS-CoV-2 [9]. AAT might play a role in the pathogenesis of COVID-19 as it inhibits the protease TMPRSS2 and thus restricts the entry of SARS-CoV-2 into cells [10]. Furthermore, AAT is essential for maintaining various physiological functions such as coagulation, immunomodulation, and inflammation regulation [4, 11, 12].

Apart from the physical symptoms associated with COVID-19 infection, the pandemic has also had a marked impact on mental health globally, with a significant rise in anxiety, stress, and depression due to quarantine measures and the risk of infection [13, 14]. The implementation of social distancing measures during the pandemic has exacerbated the impact on the quality of life (QoL) for individuals with AATD by imposing limitations on their daily activities [15]. Additionally, individuals with AATD, particularly those with poor lung function, already face an increased risk of developing mental illnesses [16]. The effect of the COVID-19 pandemic on the well-being of individuals with AATD remains unclear, highlighting the need to investigate this area. The aim of our study was to investigate the impact of the COVID-19 pandemic on the psychological well-being of individuals with AATD. Specifically, we aimed to identify sociodemographic and disease-related factors that contribute to stress-related symptoms during the pandemic and explore the potential mediating role of SGRQ in the relationship between poor lung function and stress-related symptoms.

## Methods

### Patients and study design

The study utilized the “German Register for Alpha-1 Antitrypsin Deficiency,“ which is a comprehensive database established in 2004 and operated by the Department of Internal Medicine V at the University Hospital of Saarland. The study included only individuals aged 18 years and above. The register contains a wide range of sociodemographic and disease-related parameters such as lung function data, health-related quality of life (HRQoL), therapy and course data, and exacerbation histories [17, 18]. In addition, a follow-up data collection from 2018 includes detailed information on various comorbidities and HRQoL scoring. Apart from the general analysis using the registry entry data, we conducted a separate analysis using data from follow-up examinations, including comorbidities and HRQoL scoring. This subgroup, referred to as the “expanded data collection” group, allowed for a more comprehensive analysis. To collect information on the impact of COVID-19, a questionnaire was sent to all individuals with a register entry, gathering data on COVID-19 diagnostic testing, contact reduction measurements, SARS-CoV-2 infection symptoms, changes in well-being (stress, anxiety, grief and dependency), and health status. Statistical analysis combined this data with clinical parameters. The study received approval from the ethics commission of the Landesärztekammer des Saarlandes (62/20), and all participants provided written consent.

### Data analysis

Participants included in the study were those who completed a COVID-19 questionnaire between August 2020 and December 2020, resulting in a total of 358 participants. Due to partially incomplete answers on the questionnaires, there were missing values in certain areas of the datasets.

The statistical analysis of the study was conducted with SPSS software (version 27.0) and the PROCESS macro (version 4.0) developed by Preacher and Hayes for mediator analysis within regression analysis [19]. Statistical significance was considered at a p-value of < 0.05. Mean ± standard deviation represented continuous variables, while absolute frequencies (%) were used for nominal data. The study employed a binary approach to investigate the relationship between stress-related symptoms and health status by utilizing correlative and regression analyses. The questionnaire responses were categorized into two options, namely “yes” and “no” for stress-related symptoms, and “unchanged” and “worse” for health status, allowing for a focused examination of these variables. To assess the impact of various factors on the experience of negative emotions, an ordinal logistic regression analysis was conducted. The variable “negative emotions” was measured on an ordinal scale, with higher scores indicating a greater intensity of negative emotions. The items “anxiety,“ “grief,“ and “dependency” were combined into this variable to enhance clarity and provide a more comprehensive understanding of the overall negative emotional state. Subsequently, a multiple regression analysis was conducted for the respective binary and ordinal regression models.

## Results

### Baseline characteristics

The study analyzed a total of 358 subjects with PiZZ, PiSZ, or other allele variants, with 53.6% of them being male. The overall cohort was based on registry entry data, which included a wide range of sociodemographic and disease-related parameters such as lung function, SGRQ score, therapy and course data, and exacerbation histories. At baseline, the mean FEV_1_ (l) was 2.0 ± 1.0, and the mean SGRQ total score was 39.4 ± 20.0.

In addition to analyzing the overall cohort, a follow-up examination was conducted in 2018, which included a more detailed assessment of various comorbidities and HRQoL scoring. This data was analyzed separately from the registry entry data and referred to as the “expanded data collection” group. Upon examination of the follow-up data, there were only slight differences in FEV_1_ (l) (1.6 ± 0.8) and SGRQ total score (45.2 ± 20.2) compared to the overall cohort. Table 1 summarizes the baseline data of the two cohorts.

**Table 1:** Baseline characteristics of the overall cohort and the cohort with expanded data collection

Characteristics	Overall cohort	Expanded data collection
	Mean ± SD (n)/ n (%)	Mean ± SD (n)/ n (%)
Sex (m)	192 (53.6%)	99 (53.5%)
Age (years)	63.72 ± 12.02 (355)	63.95 ± 10.52 (183)
BMI (kg/m^2^)	24.87 ± 5.23 (352)	24.55 ± 4.43 (177)
Non-smoker	110 (30.7)	73 (39.5)
Ever smoker	245 (68.4%)	123 (66.5%)
Pack-years	18.71 ± 14.17 (222)	16.87 ± 13.77 (111)
FEV_1_ (l)	2.01 ± 0.96 (307)	1.58 ± 0.82 (163)
FEV_1_ (%)	61.60 ± 26.58 (301)	55.22 ± 25.58 (151)
TLCO (mmol/min/kPa)	4.63 ± 3.12 (215)	4.35 ± 2.10 (71)
TLCO (%)	56.48 ± 25.28 (157)	47.20 ± 21.01 (54)
SGRQ total score	39.37 ± 20.04 (318)	45.15 ± 20.19 (159)
CAT score		17.28 ± 7.70 (167)

### Impact of the COVID-19 pandemic on prevalence of stress and anxiety among AATD patients

**Fig. 1:**
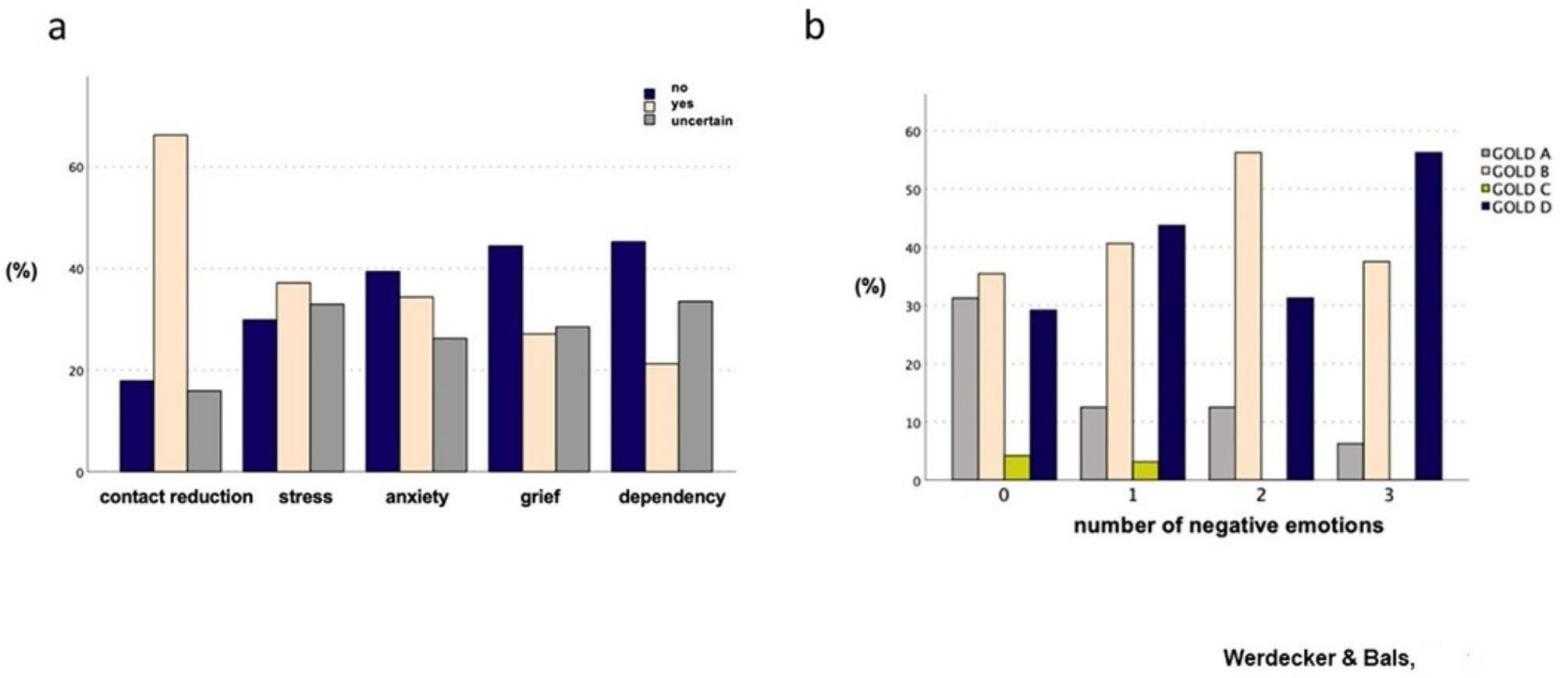
Impact of the COVID-19 pandemic on well-being. (**A**) Frequency distribution of specific stress categories in percent in the overall cohort. (**B**) Abundancy of negative emotions dependent in the ABCD GOLD classification (%) (n = 133)

The prevalence of COVID-19 infections among AATD patients in our sample was 0.6% (n = 2). Descriptive analysis of the COVID-19 questionnaire revealed that stress was reported by 37.2% (n = 133) of participants, while anxiety was reported by 34.4% (n = 123). The correlation analysis of various stress-related symptoms demonstrated a highly significant association (p < 0.001). There was a significant correlation between negative emotions, stress (Chi^2^ = 282.5; Cramer’s V = 0.6***) and contact reduction (Chi^2^ = 195.7; Cramer’s V = 0.5***). Figure 1a displays the frequencies of psychological effects of the pandemic situation.

### Exploring the relationship between lung function and psychological consequences

The correlation analysis and simple regression analysis revealed a relationship between FEV_1_ (%) and stress-related symptoms, including negative emotions, stress, and decline in health status (r_p_ = -0.121* to r_sp_ = -0.258**; OR = 0.98 to 0.99). However, after adjusting for sociodemographic and clinical characteristics in multiple regression analysis, no significant predictive power was found. To further explore this relationship, the impact of SGRQ on the association between FEV_1_ (%) and stress-related symptoms was examined (Tables 2, 3 and 4).

**Table 2:** Ordinal regression analysis of predictor variables for heightened negative emotion perception (multiple regression with n = 192). * p < 0.05; ** p < 0.01; *** p < 0.001; ∫: expanded data collection; ORs s are adjusted for variables in multiple; B = regression coefficient, OR = odds ratio, 95% CI = 95% confidence interval, p = significance

	Simple regression			Multiple regression		
Variables	B	OR (95% CI)	p	B	OR (95% CI)	p
Age	-0.016	0.984 (0.97-1.00)	0.079	-0.030	0.971 (0.95-1.00)	0.029*
Sex (male)	-0.615	0.541 (0.35–0.83)	0.005**	-0.705	0.494 (0.28–0.86)	0.012*
FEV_1_ (%)	-0.020	0.981 (0.97–0.99)	< 0.001***	-0.010	0.990 (0.98-1.00)	0.169
SGRQ total score	0.021	1.021 (1.01–1.03)	< 0.001***	0.011	1.011 (0.99–1.03)	0.254
Chronic bronchitis	0.583	1.791 (1.13–2.83)	0.013*	0.419	1.521 (0.85–2.71)	0.156
Bronchodilatation	0.736	2.088 (1.20–3.63)	0.009**	0.201	1.223 (0.52–2.87)	0.643
Occupation	0.311	1.365 (0.88–2.12)	0.165			
CAT score ∫	0.063	1.065 (1.02–1.11)	0.002**			
Mental illness ∫	0.961	2.614 (1.07–6.40)	0.036*			

**Table 3:** Logistic regression analysis of predictor variables for stress (multiple regression with n = 181). p < 0.05; ** p < 0.01; ORs s are adjusted for variables in multiple; abbreviations: B = regression coefficient, OR = odds ratio, 95% CI = 95% confidence interval, p = significance

	Simple regression			Multiple regression		
Variables	B	OR (95% CI)	p	B	OR (95% CI)	p
Age	-0.019	0.982 (0.96-1.00)	0.111	-0.039	0.961 (0.93–0.99)	0.013*
Sex (male)	-0.404	0.668 (0.40–1.12)	0.123			
FEV_1_ (%)	-0.017	0.984 (0.97–0.99)	0.002**	-0.001	0.999 (0.98–1.02)	0.908
SGRQ total score	0.018	1.018 (1.01–1.03)	0.009**	0.027	1.027 (1.01–1.05)	0.011*
Broncho-dilatation	0.839	2.314 (1.19–4.51)	0.014*	0.175	1.192 (0.46–3.08)	0.717

**Table 4:** Logistic regression analysis of predictor variables for deterioration of health status (multiple regression with n = 95). * p < 0.05; ** p < 0.01; ∫: expanded data collection; ORs s are adjusted for variables in multiple; abbreviations: B = regression coefficient, OR = odds ratio, 95% CI = 95% confidence interval, p = significance

	Simple regression			Multiple regression		
Variables	B	OR (95% CI)	p	B	OR (95% CI)	p
FEV_1_ (%)	-0.018	0.982 (0.97-1.00)	0.009**	-0.004	0.996 (0.97–1.02)	0.724
SGRQ total score	0.022	1.022 (1.01–1.04)	0.006**	0.031	1.032 (0.99–1.07)	0.118
Chronic bronchitis	0.806	2.238 (1.25–4.02)	0.007**	0.370	1.448 (0.46–4.53)	0.525
Exacerbations in the prior 2 years	0.821	2.273 (1.29–3.99)	0.004**	0.709	2.031 (1.03–4.02)	0.042*
CAT score ∫	0.064	1.066 (1.01–1.13)	0.030*			

A total of 133 participants were categorized by the COPD GOLD group system based on their current exacerbation history and CAT score. The distribution among the GOLD groups was as follows: GOLD A (18.8%, n = 25), GOLD B (42.1%, n = 56), GOLD C (2.3%, n = 3), and GOLD D (36.8%, n = 49). The number of negative emotions in AATD patients during the COVID-19 pandemic was found to be significantly correlated with increasing GOLD stage (r_sp_ = 0.211; p < 0.026) (Fig. 1b).

Additionally, a higher number of exacerbations in the past positively correlated with a decline in health status (Cramer’s V = 0.269*). Multiple regression analysis confirmed this result, showing that an increased exacerbation frequency (1-2x, 2-4x, >4x) led to a 2.03-fold increase in the chance of health status decline (OR = 2.03; 95% CI: 1.03–4.02; Table 4).

### The impact of SGRQ and CAT scores on mental well-being

The SGRQ total score exhibited a positive correlation with negative emotions, stress, and deterioration of health status. Each one-point increase in the SGRQ total score corresponded to a 1–2% increase in the chance of experiencing these effects (Tables 2, 3 and 4). After controlling for potential confounders, the regression models demonstrated significant predictive power of SGRQ total score for stress symptoms (Table 3).

**Fig. 2:**
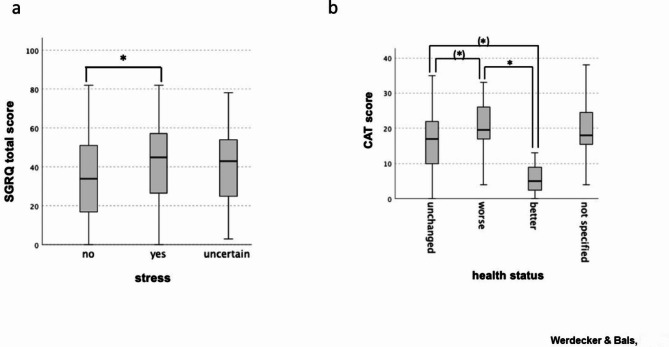
Stress or health status are associated with SGRQ and CAT quality of life instruments. (**A**) Group comparison of SGRQ total score by stress using Kruskal-Wallis test (n = 318); represented as boxplots; adjusted significance after Bonferroni correction: *p < 0.05. (**B**) Group comparison of CAT score by health status using Kruskal-Wallis test (n = 167); represented as boxplots; adjusted significance after Bonferroni correction: *p < 0.05 (significance without correction)

Similarly, the CAT score showed significant predictive power for increased negative emotions and deterioration of health status. Each higher point value on the CAT score was associated with a 7% increase in the chance of experiencing these outcomes (Tables 2 and 4). Furthermore, a group comparison of SGRQ and CAT scores based on response options was conducted for “stress” and “health status” (Fig. 2a, b).

### The SGRQ total score mediates the association between FEV_1_ (%), stress and deterioration of the health status

**Fig. 3:**
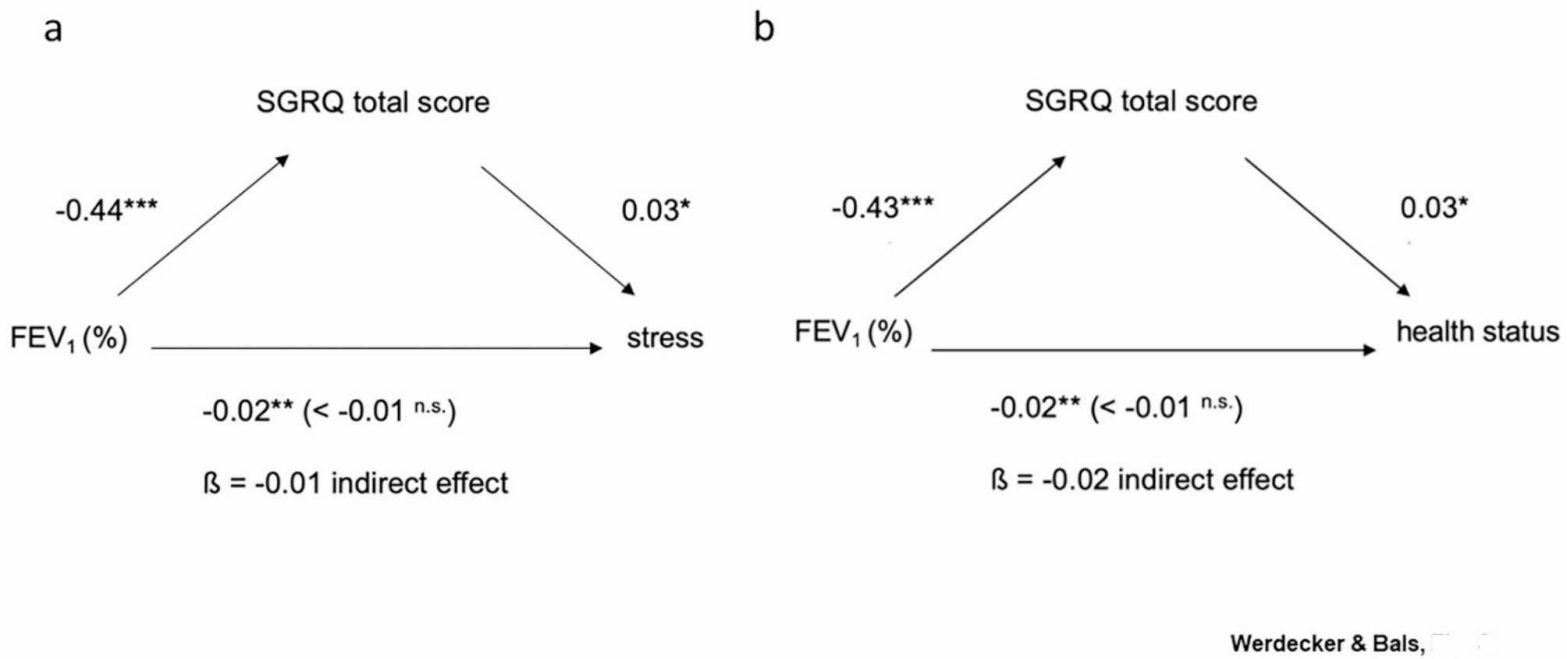
QOF as measured by the SGRQ mediates these effect of low FEV_1_ on stress or health status. (**A**) Simple mediation model of the relationship between FEV_1_ (%) and stress mediated by SGRQ total score (n = 183); covariates: sex, age. n.s. = not significant; *p < 0.05; **p < 0.01; ***p < 0.001. (**B**) Simple mediation model of the relationship between FEV_1_ (%) and deterioration of health status mediated by SGRQ total score (n = 241); covariates: sex, age. n.s. = not significant; *p < 0.05; ***p < 0.001

Mediator analysis showed that SGRQ total score influences the relationship between FEV_1_ (%) and stress. Furthermore, the relationship between FEV_1_ (%) and deterioration in health status was also mediated by SGRQ total score. When the mediator (SGRQ total score) was included in the analysis, the direct association between FEV_1_ (%) and stress symptoms or health status deterioration became non-significant, indicating a complete mediation effect. Therefore, a lower lung function does not lead to the occurrence of stress or deterioration of health status, but an effect intervened by SGRQ total score is responsible for the positive relationship (Fig. 3a). Furthermore, the mediation analysis revealed that the SGRQ total score played a mediating role in the relationship between FEV_1_ (%) and the deterioration of health status (Fig. 3b).

### Young female individuals with psychological pre-conditions suffer from the strongest emotional changes

Correlation analysis revealed a significant relationship between age and “negative emotions” (r_sp_ = -0.120*), indicating that as age increases, the experience of negative emotions tends to decrease. Additionally, there was a positive correlation between employment and “negative emotions” (r_sp_ = 0.193*), suggesting that employed individuals may experience higher levels of negative emotions. To further investigate the relationship between age and employment, we conducted a correlation analysis, revealing a significant association between these variables (r_pb_ = -0.351***).

As age increases by one year, there is a 3% decrease in the chance of experiencing heightened negative emotions (OR = 0.97; 95%-CI: 0.95-1.00; Table 2). Furthermore, multiple regression analysis showed that each additional year of age was associated with a 4% decrease in the odds ratio for stress (OR = 0.96; 95%-CI: 0.93–0.99; Table 3).

Subsequent analysis of the follow-up data focused on the impact of comorbidities on well-being. While no significant relationships were found between various comorbidities (e.g. cardiovascular diseases, musculoskeletal diseases) and stress-related symptoms, a strong association was observed with mental illness. Individuals with mental illness had a 2.6-fold increased chance of experiencing heightened negative emotions (OR = 2.61; 95%-CI: 1.07–6.40; Table 2) and a 5.6-fold increased chance of AATD rising in importance (OR = 5.61; 95%-CI: 1.74–18.07). Mental illness emerged as one of the strongest predictors in our analysis.

To evaluate potential interactions between sex and distress symptoms, group analyses were conducted using regression analysis. The results revealed a statistically significant relationship between gender and negative emotions (Cramer’s V = 0.188*). Men had a 51% reduced risk (OR = 0.49; 95% CI: 0.28–0.86) of experiencing further negative emotions as compared to females. Specifically, men had a significant influence on experiencing “grief” (OR = 0.41; 95% CI: 0.24–0.69) and “anxiety” (OR = 0.57; 95% CI: 0.35–0.94), with a 43% lower chance of developing anxiety symptoms and a 59% lower chance of experiencing grief. Moreover, the gender-specific analysis revealed that the effect of chronic bronchitis on health deterioration was more pronounced in women (OR = 3.89; 95% CI: 1.54–9.82).

## Discussion

The main finding of this investigation is the high prevalence of negative emotions, such as stress and anxiety, and a decline in health status among individuals with a rare lung disease during the early months of the COVID pandemics. These negative emotions and health deterioration are correlated with reduced lung function (FEV_1_ (%)) and impaired quality of life (SGRQ total score). Furthermore, QOL as determined by the SGRQ total score acts as a mediator between lung function and the experience of stress and declining health. Additional risk factors identified in this study include a history of exacerbations, comorbid mental illness, younger age, and female sex. The findings also highlight a link between negative emotions, perceived stress and reduced social interaction. These results underscore the importance of addressing the psychological impact of rare lung diseases and implementing targeted interventions to improve the well-being of affected individuals.

Studies have shown that the pandemic situation leads to increased psychological reactions, particularly manifested in anxiety, depression and stress [14]. Individuals with chronic illnesses, including lung disease, are vulnerable to these negative psychological effects [20]. However, our study found no significant difference in the prevalence of anxiety symptoms between the AATD cohort and the general population (34.4% vs. 34.5%) [21]. Interestingly, the present data suggests that younger individuals are more likely to experience anxiety symptoms and stress. Negative emotions were found to be significantly associated with employment, indicating that negative impacts on work life may contribute to the heightened psychological burden experienced by younger individuals. This finding aligns with previous studies that have also highlighted the negative influence of social media on the mental well-being of younger individuals [22]. The underlying causes of anxiety in AATD patients during the pandemic may be multifaceted. Our analysis revealed a relationship between negative emotions and reduced contact, suggesting that increased feelings of loneliness due to reduced interpersonal communication during quarantine measures may contribute to anxiety, as observed in other analyses [23]. Additionally, regression analysis identified female gender and psychological comorbidity as strong and significant predictors of negative emotions and anxiety, consistent with previous analyses that have reported similar findings [24].

Additionally, our study findings revealed that individuals who reported stress not only had lower FEV_1_ (%), but also generally experienced a lower health-related quality of life (SGRQ). Mediation analyses indicated the significant role of SGRQ, suggesting that individuals with advanced stages of AATD lung disease may face an increased risk of experiencing stress and a deterioration in their health status. Moreover, it is important to consider that a high HRQoL may play a crucial role in mitigating psychological impacts. Existing evidence from studies conducted on the general population has shown a significant association between HRQoL and anxiety levels during the pandemic [25]. This suggests that individuals with better HRQoL may experience lower levels of anxiety. Other researchers have pointed out that poor lung function in AATD patients can potentially contribute to the development of depression, considering the impact of disease-related symptoms and social isolation resulting from impaired lung function [15, 25, 26]. Our study provides evidence that individuals with AATD and a history of frequent exacerbations experienced notable psychological impacts, with stress being particularly prominent. These results are consistent with previous studies that have reported a similar association between psychological impacts during the COVID-19 and a complex medical history [13].

The study has both limitations and strengths. One limitation is the restricted data collection period of four months, without data collected before or after, which limits the comprehensive assessment of the psychological response. Another limitation is the potential selection bias due to the inclusion of participants based on their motivation to participate. Additionally, most of the data was obtained through self-assessment questionnaires, which may diminish the data quality and introduce the possibility of overestimation or underestimation of the true effects. However, the study boasts strengths in the comprehensive regional distribution of participants and the incorporation of a broad range of clinical parameters, enhancing the generalizability of the findings.

In conclusion, poor lung function (FEV_1_%) and low health-related quality of life (SGRQ) have been found to be associated with negative psychological effects in individuals with AATD during the early phase of the COVID-19 pandemic. Our study identified a mediation pattern, confirming that SGRQ plays a mediating role in the relationship between FEV_1_ (%) and perceived stress, as well as the deterioration of health status. Additionally, a higher number of exacerbations was positively linked to a decline in health status. Younger age, female gender, and mental illness were identified as predictors of negative emotions and anxiety symptoms. These findings emphasize the importance of addressing and managing negative psychological outcomes in patients with rare diseases during pandemic conditions.

## Data Availability

The datasets can be accessed through the authors.
